# Immune and non-immune cell subtypes identify novel targets for prognostic and therapeutic strategy: A study based on intratumoral heterogenicity analysis of multicenter scRNA-seq datasets in lung adenocarcinoma

**DOI:** 10.3389/fimmu.2022.1046121

**Published:** 2022-11-22

**Authors:** Tianyu Fan, Jian Lu, Delei Niu, Yue Zhang, Bin Wang, Bei Zhang, Zugui Zhang, Xinjiai He, Nan Peng, Biao Li, Huilong Fang, Zheng Gong, Li Zhang

**Affiliations:** ^1^ The Department of Immunology, College of Basic Medicine, Qingdao University, Qingdao, Shandong, China; ^2^ Department of Orthopaedics, Suzhou Science and Technology Town Hospital, Suzhou, Jiangsu, China; ^3^ The Department of Pathogenic Biology, College of Basic Medicine, Qingdao University, Qingdao, Shandong, China; ^4^ Value Institute, Christiana Care Health System, Newark, DE, United States; ^5^ Department of Radiation Oncology, The Affiliated Hospital of Qingdao University, Qingdao, Shandong, China; ^6^ Department of Pathogenic Biology and Immunology, Xiangnan University, Chenzhou, Hunan, China; ^7^ Sino-Cell Biomed Institutes of Medical Cell and Pharmaceutical Proteins, Qingdao University, Qingdao, Shandong, China; ^8^ Department of Basic Medicine, Xiangnan University, Chenzhou, Hunan, China

**Keywords:** lung adenocarcinoma (LUAD), TCGA, scRNA-seq, immunotherapy, microenvironment

## Abstract

Lung adenocarcinoma (LUAD) is the most common type of lung cancer and the leading cause of cancer incidence and mortality worldwide. Despite the improvement of traditional and immunological therapies, the clinical outcome of LUAD is still far from satisfactory. Patients given the same treatment regimen had different responses and clinical outcomes due to the heterogeneity of LUAD. How to identify the targets based on heterogeneity analysis is crucial for treatment strategies. Recently, the single-cell RNA-sequencing (scRNA-seq) technology has been used to investigate the tumor microenvironment (TME) based on cell-specific changes and shows prominently valuable for biomarker prediction. In this study, we systematically analyzed a meta-dataset from the multiple LUAD scRNA-seq datasets in LUAD, identified 15 main types of cells and 57 cell subgroups, and revealed a series of potential biomarkers in M2b, exhausted CD8^+^T, endothelial cells, fibroblast, and metabolic patterns in TME, which further validated with immunofluorescence in clinical cohorts of LUAD. In the prognosis analysis, M0 macrophage and T cell activation were shown correlated to a better prognosis (*p*<0.05). Briefly, our study provided insights into the heterogeneity of LUAD and assisted in novel therapeutic strategies for clinical outcome improvement.

## Introduction

Lung cancer is the leading cause of cancer death globally, and the most prevalent subtype of lung cancer is lung adenocarcinoma (LUAD) (1). Despite the great endeavors in traditional and complementary treatments, the clinical outcomes are still not satisfactory (2, 3). The process of oncogenesis and cancer development is influenced by the tumor microenvironment (TME) and the tumor cells through mutual and dynamic crosstalk. The TME is consisted of immune cells (like lymphocytes, macrophages, and microglia), tumor stromal cells (including stromal fibroblasts and endothelial cells), the non-cellular components of the extracellular matrix, and the tumor cells (4, 5). And a growing number of therapeutic strategies were focused on TME, such as cancer-associated fibroblasts (CAFs), tumor-associated macrophages (TAMs), and CTLA-4/PD-1/PD-L1 immune checkpoints (6–8). Due to the heterogeneity of LUAD, patients given the same treatment regimen had different responses and clinical outcomes. Therefore, the identification of targets based on intratumoral heterogenicity analysis is extremely crucial for novel and precise therapeutic strategies in LUAD.

The TME was so complex that essential to study further for clinical outcome improvement in LUAD (5, 9). RNA sequencing (RNA-seq) had already been independently made to predict the prognosis-related genes and assessment their correlation with clinical outcomes in TME. Reports showed immune subtypes in LUAD TME with prognostic and therapeutic implications (10). Currently, single-cell RNA sequencing (scRNA-seq) is widely used to identify biomarkers in diagnosing, treating patients, and studying the heterogeneity in TME. Intratumoral heterogeneity could be analyzed by scRNA-seq at the cell-type level; in contrast, the conventional bulk RNA-seq obtained the average expression of genes, and difficult to study the heterogeneity in TME. And due to the cancer heterogeneity, patients’ response is different significantly to certain treatment. Recently the heterogeneity of stromal cells and tumor-infiltrating immune cells associated with immunotherapy responses had been widely reported (11). The knowledge about the mechanism responsible for the LUAD heterogeneity was still poor, even if many scientists were devoted to elucidating these issues. To date, although numerous scRNA-seq studies on LUAD had been reported, most of these studies were limited by small sample sizes and imperfect controls. In this study, we constructed a meta-dataset from multiple scRNA-seq datasets (GSE131907, GSE134355, and GSE148071) and analyzed the immune and non-immune diversity clusters in TME, dug out targets for treatment, and assessed their prognostic value in LUAD. Briefly, our study systemically provided insights into the heterogeneity of LUAD and assisted in precise and novel strategies for prognosis and target treatments.

## Materials and methods

### Acquisition of data

The expression matrix and patients’ clinical information from three datasets (GSE134355, GSE131907, and GSE148071), which contained 19 normal and 53 LUAD samples, were downloaded from the GEO database (https://www.ncbi.nlm.nih.gov/geo/). The GSE134355 dataset was generated from Illumina HiSeq X Ten and GPL20795 platform. The GSE131907 dataset was generated from Illumina HiSeq 2500 and GPL16791 platform. The GSE148071 dataset was generated from Illumina HiSeq X Ten and GPL20795 platform.

### QC and cell type recognition

Using Seurat (version 4.2.0) performed the QC process (12). We excluded cells with a mitochondrion-derived UMI count of more than 10% or less than 200 UMIs as low-quality cells. ScaleData was used to remove the influence of UMI counts and mitochondrion-derived UMI counts. The quality control (QC) process used the Seurat R package. The QC parameter setup and candidate cells filter by the following criteria: nFeatue_RNA>200 & <7000 percent.mt<25. We also used the VlnPlot function of the Seurat package to generate the QC figure (**Figure S1A**) and show the value (nFeaure_RNA, nCount_RNA, mito_RNA, and ribo_RNA) after QC. The Harmony R package was used to correct batch effects (**Figure S1A** down). The next step was using Seurat’s FindClusters function (resolution = 1.1) to identify the main cell clusters and utilizing 2D tSNE or UMAP to visualize (13). Currently, for data dimension reduction, these algorithms were most commonly used. The downstream analysis did not perform on the primary cell cluster due to the difference in the cell cycle. Each cell cluster’s markers were listed using the FindAllMarkers function. Based on the CellMarker database, the major cell types were identified and annotated (14).

### Immune checkpoint gene analysis

To represent the gene expression levels in different cell clusters, we calculated the mean normalized immune checkpoint gene expression levels from cell clusters and then normalized them into row Z scores. The immune checkpoint gene heatmap analyses were performed using the ComplexHeatmap R package. We used the ComplexHeatmap::pheatmap function and set-up parameters: scale = “row” to calculate the Z-score of genes mRNA expression level, then the heatmap was colored according to this Z-score.

### Core transcription factors regulatory network analysis

The core regulatory transcription factors and their regulatory network were predicted using the R package SCENIC. The R software (version 4.0.2) was used to reconstruct the regulatory networks and display the transcriptional characterization (15). The value of the area under the curve (AUC) was estimated by SCENIC, then the Limma was used to identify differences in AUC among cell clusters or between normal and tumor-derived cells of each module. Regulators were investigated further through the adj. *p* val< 0.01.

### Pseudotime trajectory analysis

We used Monocle 2 for single-cell trajectories analysis, an R package developed by Qiu et al. (16). We revealed the alteration of the CD8^+^ T cell during tumor-educating. We optimized the input parameters as following: mean expression ≥ 0.125, num_cells_expressed ≥10, and in the differentialGeneTest function *q*val < 0.01 was considered as significant. 2D tSNE plots were used to visualize the trajectories and plot_pseudotime_heatmap was used for constructing the dynamic expression heatmaps.

### InferCNV

The InferCNV R package was used for CNV analysis. Through InferCNV, you could visualize CNV in cells according to RNA-Seq expression data. Genes were analyzed, including their relative expression levels and chromosomal locations to estimate CNVs (17, 18). Cell types were initially classified by using the Seurat package. CNV was calculated for all euchromosome types using InferCNV. For 10× Genomics single-cell data, the cut-off value was 0.1.

### Functional enrichment analysis

The FindMarkers function of Seurat was used to identify DEGs. The cut-off thresholds were adj. p value <0.01 and fold change (FC) >1.5. Then, GO enrichment analysis was carried out using clusterProfiler (19) on these DEGs. An enrichment adj. *p* val <0.05 was considered statistically significant.

Gene set was enrichment in each specific cell cluster and was performed by GSEA analysis. Only gene sets were significantly enriched with false discovery rate (FDR) *p* values <0.05 and nominal *p* values <0.05.

The GSVA package was adopted for performing gene set variation analysis (GSVA) and using default configuration parameters. The cytokine pathway gene sets or 50 hallmark gene sets were downloaded from the GSEA molecular signature database.

### Cell-cell communication analysis

The CellChat R package provided a means for analyzing cell-to-cell communication at the molecular level through R software. First, 16 types were clustered from 24,550 single cells as described above. Analysis of 16 subclusters and major cell types was carried out using CellChat to examine molecular interaction networks. The CellChat estimated the ligand-receptor pairs. And the result with *p* values <0.05 would be retained for evaluating the cluster-by-cluster analysis.

### Correlation to public datasets

The deconvolution analysis was performed on the integrated bulk RNA-seq data (TCGA-LUAD) against our scRNA-seq dataset, which was conducted using the BisqueRNA package with default settings (20). We labeled our cells into 15 categories, including macrophages, B cells, NK cells, DC cells, fibroblasts, CD8^+^ T cells, CD4^+^ T cells, epithelial cells, endothelial cells, Mast cells, smooth muscle cells, neutrophils, plasma cells, and myeloid. The group comparisons were then made using the composition of deconvolution cell types in each bulk sample. The Cox regression analysis to assess the prognostic value of different cell clusters. Visualization of Cox regression results was achieved using Z scores. To determine if the relative abundance of cell clusters’ dynamical alteration was associated with the LUAD progression (WHO clinical stage).

### Immunofluorescence assay on human LUAD tissue

Sections of tissue containing 25 pairs of para-tumors and tumors were obtained from the Affiliated Hospital of Qingdao University (NO: QDU-HEC-2022227). Patient information was listed in **Figure S2**. The immunofluorescence was performed on the same type of tissue sections for the analysis to be consistent.

The antibodies were applied to validate the specific markers were identified in this study as follows: anti-FGFBP2 (R&D system, catalog. AF9349-SP), anti-PRF1(abclone, catalog. A0093; RRID: AB-2749981), anti-CD163 (abclone, catalog. A8383; RRID: AB-10687227), anti-ATP5F1E (abclone, catalog. A7645; RRID: AB-2768505), RRID: AB-853002), anti-LAG3 (Abcam, catalog. 209236; RRID: AB-2162568), anti-CLDN4 (Abcam, catalog. ab53156), anti-CLDN1 (Abcam, catalog. ab211737), anti-ACTA2 (Abcam, catalog. ab264014), anti-RALA (Abcam, catalog. ab236314). Data analysis was performed with GraphPad Prism (version 9) software.

### Flow cytometry

We mechanically separated and enzymatically digested the collected tumor tissue to prepare a single-cell suspension (collagenase (Solarbio), DNase I (Solarbio), and Dispase I (Solarbio); prepared in DMEM) at 37°C for 1 h. Filter with a 40 μm cell strainer. The lymphocytes are then isolated with a tumor-infiltrating lymphocyte isolation solution kit. The isolated cells are washed once with PBS at 4°C and stained with antibodies from 3 different channels for 1 h. The antibodies were applied as follows: anti-CD8 (Abcam, catalog. 233300; anti-TIM3(Abcam, catalog. ab28522), anti-PD 1 (Abcam, catalog. ab52587). Data analysis was performed with FlowJo (version 10) software.

### Statistical analysis

Our analysis was conducted using the R software and package, Spearman correlation analysis was performed, and heatmaps and scatterplots were generated as a result. We also used the online tool GEPIA, which analyzes pan-cancer tissue-specific expression. The immunofluorescence results were statistically analyzed by ImageJ software and the flow cytometry results were statistically analyzed by CytExpert software. It was considered statistically significant if *p* < 0.05.

## Results

### The LUAD cell types and normal lung tissues

**Figure 1:**
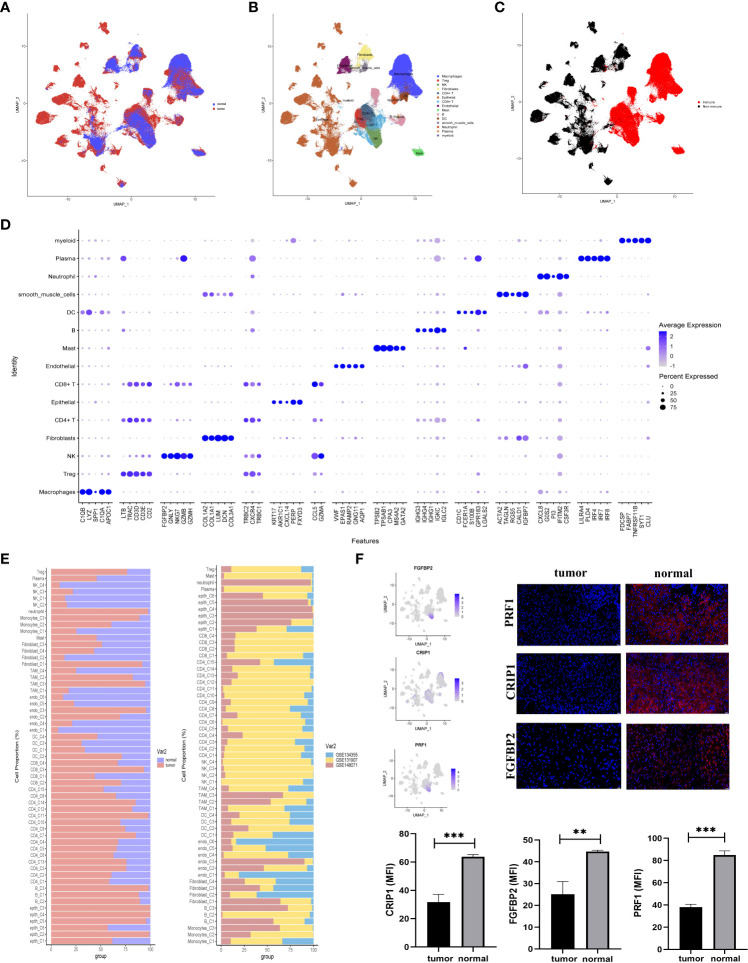
Comprehensive scRNA-seq analyses of cells derived from LUAD or normal tissues. **(A–C)** UMAP plot of single-cell transcriptome data with cells colored based on **(A)** tissue type origin (normal or tumor), **(B)** 15 major cell types, and **(C)** the immune cells or the non-immune cells. **(D)** The dot plot showed the top five markers of 15 major cell types. **(E)** The 57 subsets were identified in this study: the relative proportion of cells derived from the normal or tumor specimens (left); and the relative proportion of cells derived from each of the three different datasets (right). **(F)** The expression status of three normal-specific proteins, immunofluorescence and statistical analysis of RPF1, CRIP1, and FGFBP2 in tissue sections. The scale bar represented 20 µm, **p<0.01, ***p<0.001.

Three GEO datasets (GSE134355, GSE131907, GSE148071) were involved in this study. Of these, the dataset (GSE134355) originated from normal lung tissues, the dataset (GSE148071) was tumor-derived cells, and the dataset (GSE131907) originated from both normal and tumor-derived cells (**Figure 1A**). A total of 15 main cell types were identified in these cells (**Figure 1B**). Eleven major immune cell types (CD45^+^) were identified, containing CD4^+^ T cell, CD8^+^ T cell, natural killer (NK) cell, B cell, regulatory T cell (Tregs), dendritic cell (DCs), plasma, myeloid, macrophages mast cell, and neutrophil, as well as the four non-immune cell types (CD45^-^), including epithelial, smooth muscle cells, fibroblasts, and endothelial cells (**Figures 1B, C**). Furthermore, the known markers mentioned in the CellMarker database were also investigated (**Figure S1B**). The differences in the cell cycle stages at the level of the single cells were not analyzed in the downstream analysis (**Figure S2A**). A bubble chart was created to visualize the top five cell-type markers (**Figure 1D**). We performed an irGSEA analysis in **Figure S2F**, in this figure, we demonstrated the situation of the top 50 signaling pathways in different cell clusters.

These major cell types were divided into two subclusters (immune and non-immune cells) to further identify their cell subclusters (**Figures S2B, C**). In total, 57 different cell clusters were identified, including 41 clusters of immune cells and 16 clusters of non-immune cells in the TME of LUAD. Several points were worth noting in **Figure 1D**. First, tumor tissues had high levels of CD4^+^FOXP3^+^ Treg cells. Second, CD8^+^ T (C3) cells were tumor-specific. Additionally, epithelial enriched in several different cell clusters and mainly existed in LUAD tissues (**Figures 1A, B, E**).

In the comparison of differentially expressed genes (DEGs) between LUAD and normal tissues, three genes (FGFBP2, CRIP1, and PRF1) were mainly expressed in normal tissues but not in tumor-derived cells (**Figure 1F**). For verification, we conducted immunofluorescence at the protein level (**Figure 1F**). The results highlighted the upregulation of FGFBP2, CRIP1, and PRF1 for potential clinical application in LUAD.

### M2b polarization in the TME of LUAD

**Figure 2:**
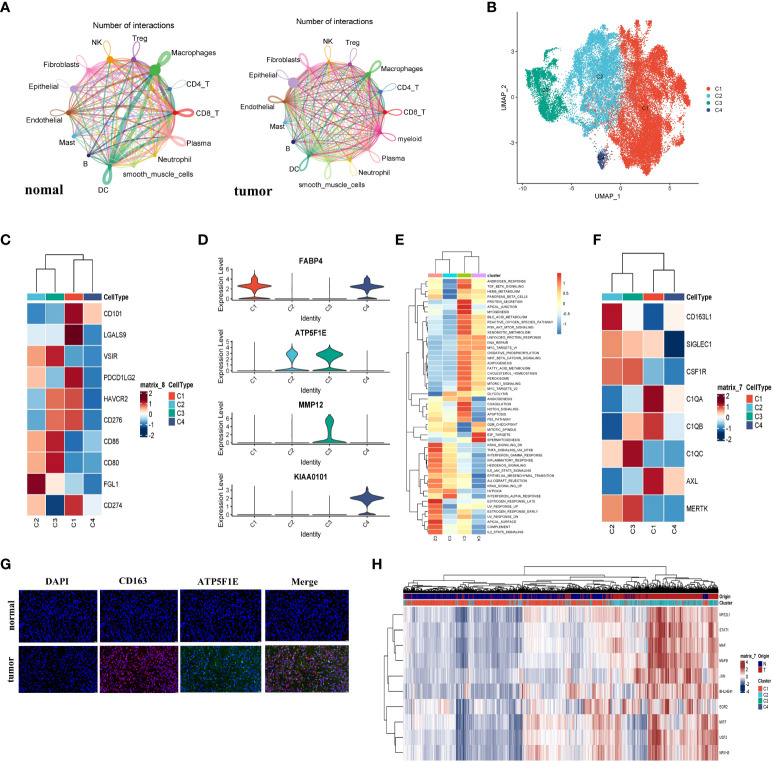
The macrophage cells demonstrated M2b polarization in LUAD. **(A)** The cell interaction network generated by CellChat; normal and tumor (left to right); the nodes size and color represented the counts of interaction; the larger size and brighter color correspond to more frequent interactions for different cell types. **(B)** The UMAP plot showed all four types of macrophage cells. **(C)** The heatmap showed the expression level of the immune checkpoint gene on macrophages and represented by a row Z score. **(D)** Violin plots of marker genes for four subgroups. **(E)** The Heatmap demonstrated the expression of the famous M2-like TAM markers. The expression level of the cell cluster was represented by a row Z score. **(F)** Immunofluorescence staining analyzed the expression of CD163 and ATP5F1E in LUAD or normal specimens. The CD163+ ATP5F1E+ macrophages exclusively appeared in tumor specimens. The scale bar represented 20µm. **(G)** The heatmap showed the differences in the activities of 50 hallmark pathways using GSVA. **(H)** The heatmap showed the differences in expression regulation by transcription factors, and the AUC scores were estimated by SCENIC.

We investigated the interaction network among the 214799 cells in the TME of LUAD. To estimate potential ligand-receptor pairs, we adopted the CellChat R package to analyze and visualize cell-cell communication molecules in normal or tumor-derived tissues. Notably, the interaction pairs between macrophages and other cells were significant revealing the macrophages with critical regulatory function in the TME (**Figure 2A**).

To investigate the heterogeneity of macrophages, we divided 45760 macrophages into four subclusters (**Figure 2B**). The cluster 1 (C1) and cluster 4 (C4) cells were mainly derived from normal tissue, while cluster 2 (C2) and cluster 3 (C3) were mainly derived from tumor tissue (**Figure S3A**). TMEs in LUAD were examined for immune checkpoint distribution. **Figure 2C** showed that the C1 cells expressed a relatively higher CD274 (PD-L1) and PDCD1LG2 (PD-L2) than other clusters. These molecules might bind to PD-1 and inhibit CD8^+^ T cell activity. Moreover, a major LAG3 ligand, FGL1 (21), was major expressed in C2 macrophages. Since C1 and C2 macrophages were more immunosuppressive than others, the cytotoxic T lymphocyte (CTL) function could be suppressed. Next, we found that cells from C1 (CD68^+^CD163^+^FABP4^+^) enriched in the TGF-β pathway from **Figure S3D**, which was characteristic of the M2a cluster. To determine whether FABP4 was associated with M2-like TAMs, we performed the spearman correlation analysis between FABP4 and other identified markers; all spearman correlation coefficients were higher than 0.3 (**Figure S3C**). In **Figure 2D**, the cells from C2 exhibited the CD68^+^CD163^+^ATP5F1E^+^ MMP12^-^ phenotype and demonstrated a high IL-10 pathway and low IL-12 pathway. The gene set variation analysis (GSVA) exhibited that the Th2-related inflammation pathways were enriched from C2 (**Figure 2E**), which were the M2b-like TAMs hallmarks, as depicted according to an earlier study (22). These results indicated that the C2 cells have an M2b-like TAMs phenotype (**Figure S3D**). In recent studies, a high level of expression of TAM markers was also observed in C2 cells (**Figure 2F**). To determine the presence of C2 cells, we did immunofluorescence and the result showed that the CD163^+^TATP5F1E^+^ macrophages were mainly enriched in LUAD tissue (**Figure 2G**). The ATP5F1E and MMP12 genes involved in the energy metabolism pathway were specifically expressed in the C3 cells (**Figure 2D**). The GSVA showed that the C3 cells could play a pro-inflammatory and antitumor role in LUAD (**Figure 2H**). This result revealed that the C3 cells tend to have an M1-like phenotype. The macrophages from C4 showed that KIAA0101 and FABP4 were preferentially upregulated (**Figure 2D** and **Figure S3B**). Combining the GSVA analysis and the above results, we inferred that C4 tended to have an M0-like TAMs phenotype. We also performed Psuedotime analysis of the macrophage cluster, and the results showed a population of M2b cells enrichment at a terminal branch of tumor tissue. Taken together, M2b (C2) and M1 (C3) were the main subgroups of macrophages in the TME in LUAD.

The SCENIC analysis demonstrated that the activity of transcription factors including STAT1, NFEIL1, MAF, MAFB, JUN, BHLHE41, EGR2, MITE, USF2, and NR1H3 was upregulated in C2 cells, while the JUND, FOSL1, FOSL2, FOS, and STAT4 transcription factors activity were downregulated (**Figure 2H**). It was reported that NFE2L1 played a vital role in the carcinogenic process (23), and EGR2 was M2-exclusive (24). Furthermore, a study on murine sarcoma also demonstrated that tumorigenesis and progression were associated with STAT1 pathway activation (22). The results supported the M2b polarization in LUAD, and also shed light on the candidate transcription factors and potential mechanisms.

### Exhausted CD8^+^ T cells enriched in the TME of LUAD

**Figure 3:**
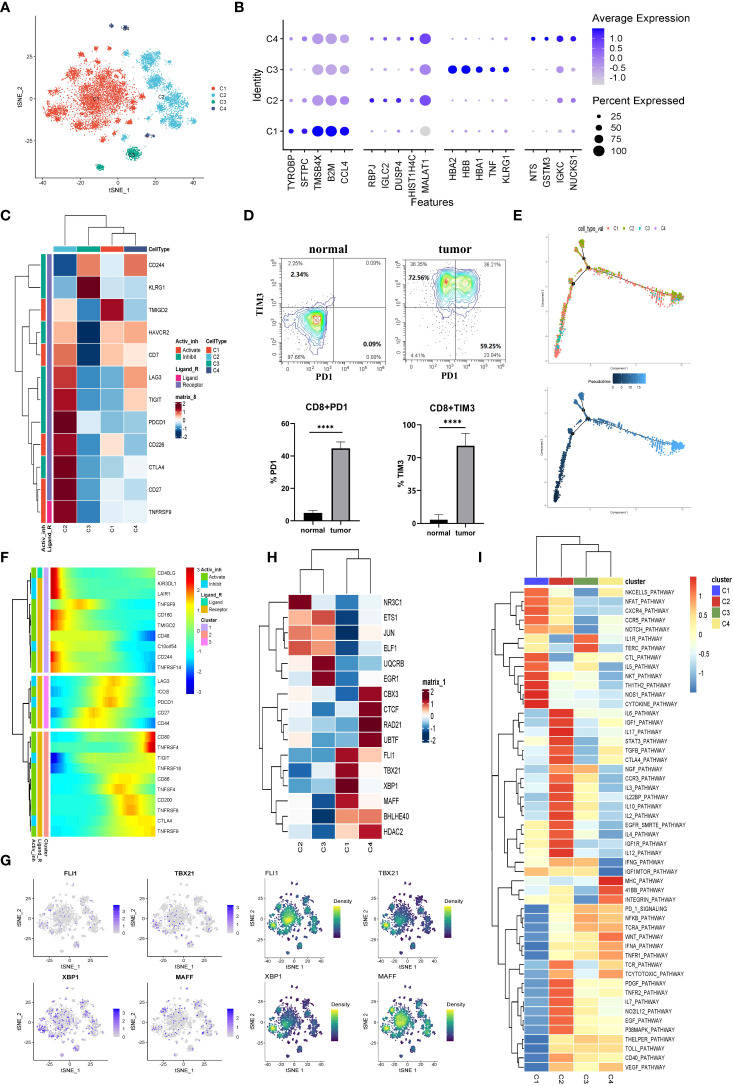
The CD8+ T cells in tumor TME preferred to be exhausted state. **(A)** The CD8+ T cells were subdivided into 4 clusters and represented on the tSNE plot. **(B)** The top five markers of the four major cell clusters were shown on the dot plot; the color represented expression level, while the sizes of dots represented abundance. **(C)** The heatmap demonstrated the downregulated or upregulated immune checkpoints in exhausted T cells. The expression level was represented by a row Z score. **(D)** The exhausted T cells in LUAD were analyzed by flow cytometry. The PD1+CD8+ and TIM3+CD8+ T cells demonstrated specifically enrichment in LUAD specimens. Gate from CD8+T cell, ****p<0.0001.. **(E)** The CD8+ T cells’ differentiation trajectory in LUAD and normal tissue and the color represented for clusters (up) or pseudotime (down). **(F)** The pseudo-heatmap showed the variation of immune checkpoint genes expression with the CD8+ T cells differentiation in LUAD, which could be subgrouped into three subcategories. **(G)** The tSNE plot represented the expression level of the indicated transcription factors (left) and the estimated AUC for these transcription factors’ activity (right). **(H)** The heatmap showed the transcription factors’ activity through SCENIC estimated AUC scores. **(I)** Heatmap of differences in activities of immune-related signaling pathways scored by GSVA.

A total of 13670 CD8^+^ T cells were analyzed in this study. And the CD8^+^ T cells were the predominant cell type in the LUAD compared with the normal tissue-derived cells. And the CD8^+^ T cells were then segregated into four subgroups. The cells from C2 (MALAT1^hi^), C3 (HBB^hi^), and C4 (IGKC^hi^) almost specially originated from tumor tissues, while C1 (TMSB4X^hi^) was almost entirely derived from normal tissue (**Figures 3A, B**, and **Figure S4A**). Furthermore, unlike the above groups, we simultaneously divided the T cells into four groups (Tn, Naive T cell; Tcm, Central Memory T cell; Tem, Effective Memory T Cell; Te, Effector T cell) and visualized them. The biomarkers expression by FeaturePlot function (**Figure S3D**) to represent these four subtype groups CD8+ T situation.

Subsequently, the immune checkpoints were examined in all the cell clusters (**Figure 3C**). The expressions of checkpoints, CTLA-4, CD27, TIGIT, PDCD1 (PD-1), LAG3, TNFRSF9, and HAVCR2 (TIM3), were upregulated in cells from C2. Based on the knowledge of their role as exhaustion markers of T cells, these data implied that C2 cells tended to be exhausted in the TME of LUAD. We then verified this phenomenon through flow cytometry. As exhibited in **Figure 3D**, the exhausted molecules were highly enriched in the tumor tissues. Currently, the treatment targets CTLA-4, PD-1, and PD-L1 as the most popular immunotherapy were wildly used in the clinic. Since the expression of CTLA-4 and PD-1 were the highest in the exhausted T cell subgroup (C2) and the C2 cells were mainly of tumor origin. Hence our data further confirmed that CTLA-4 and PD-1/PD-L1 might be significant targets for immune therapies in LUAD.

We inferred cell differentiation trajectory using Monocle 2 pseudotime analysis. And t-Distributed Stochastic Neighbor Embedding (tSNE) plot was utilized to visualize the trajectory (**Figure 3F**). Interestingly, a subgroup of CD8^+^ T cells from C1 was obtained from normal tissue and transformed into tumor-infiltrating T cells. At the terminal of the differentiation trajectory was the exhausted T cell cluster (C2) (**Figure 3E**). In this process, the immune checkpoints of promoting immune cell activation and antitumor immune responses (CD160, TNFRSF14) tended to be downregulated, while the immune checkpoints (TIGIT, TNFRSF9, CTLA-4, LAG3, PD-1) associated with exhausted T cell tended to be upregulated (**Figure 3F**). A total of three modules of DEGs were identified, and the CD8^+^ T cells were sorted into three subgroups based on their expression profile (**Figure S4B**). In exhausted CD8^+^ T cells, the cell adhesion, the ubiquitin-mediated proteolysis, and the histone modification gene set were highly enriched according to a Metascape enrichment analysis. In addition, we noted that T cell activation and cytokine production existed at the earlier stage of CD8^+^ T differentiation. These results suggested that the CD8^+^ T cells were activated in the early stages and then exhausted after continuous antigen stimulation. The TOX was the critical regulator of the differentiation of tumor-specific T cells, which also showed a constant upregulation during this process (**Figure S4C**) (25).

The SCENIC analysis was conducted to determine transcriptional activity in LUAD-specific T cells (**Figures 3G, H**). Four members (FLI1, TBX21, XBP1, and MAFF) involved in the inflammatory response, cell proliferation, and activation function were significantly activated in C1 (**Figure 3G, H**). Furthermore, the CTL pathway also was enriched in C1 (**Figure 3I**). In contrast, these transcription factors’ activity was significantly suppressed in C2, such as TGF- β (**Figures 3G–I**). Additionally, the STAT3 pathway associated with immunosuppression was upregulated in C2 cells (**Figure 3I**). These results suggested that the exhausted CD8^+^ T (C2) was intimately related to an immunosuppressive microenvironment (26). Additionally, these data provided clues for identifying new candidate transcription factors involved in dysfunctional T cells in LUAD patients.

### Extremely abnormality in the metabolism of LUAD

**Figure 4:**
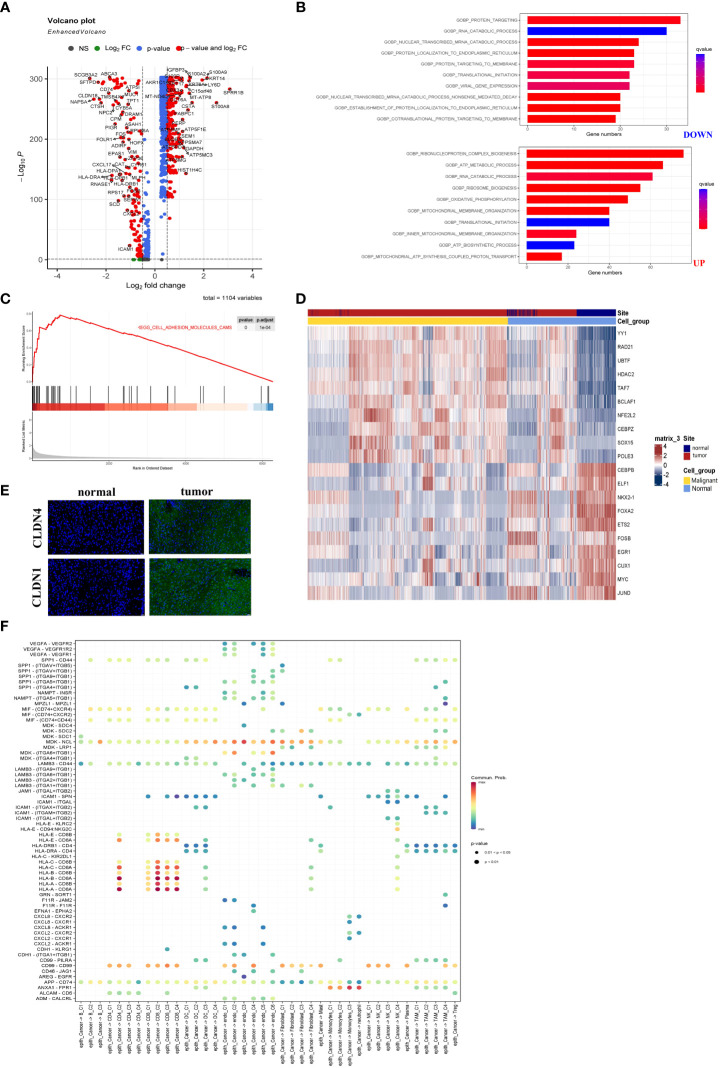
The metabolic abnormality was a specific characteristic of LUAD. **(A)** Volcano plot showed DEGs between malignant and non-malignant epithelium. Upregulated and downregulated genes (FC >2 and FDR <0.01) were colored in red. **(B)** Analyzed upregulated and downregulated DEGs using Gene Ontology. The brighter red color was considered a smaller FDR value (FDR <0.01). **(C)** The malignant epithelium was significantly enriched in the ADHESION pathway by GSEA. **(D)** Immunofluorescence staining of CLDN1 and CLDN4 in LUAD or normal tissue. CLDN1 and CLDN4 only emerged in LUAD tissues. The scale bar represented 20 µm. **(E)** The heatmap showed the transcription factors’ activity through SCENIC estimated AUC scores. The value was implicated into a row Z score. **(F)** The bubble plot showed selected ligand-receptor pairs. The CellChat R package investigated ligand-receptor interactions between malignant cells and other TME-infiltrated cell clusters.

The malignant epithelial cells and non-malignant normal epithelial cells were evaluated from scRNA-seq data using the InferCNV algorithm. The DEGs between malignant epithelial cells and non-malignant epithelial cells were identified. There were 89 DEGs, including 29 up-regulated and 60 down-regulated genes (**Table S1**). Astoundingly, the DEGs were significantly associated with energy metabolic processes, including upregulated and downregulated DEGs (**Figure 4A**). Therefore, we analyzed the upregulated and downregulated DEGs by Gene Ontology (GO) enrichment. As shown in **Figure 4B**, catabolism was enriched in malignant cells, while ATP and protein anabolism were suppressed. This result may explain the immunosuppressive properties of LUAD TME.

As we had described in **Figure 4C**, the GSEA demonstrated that the cell adhesion molecules pathway was enriched in malignant cells. **Figures S5A** and B showed that three members (CLND1, SDC1, and ALCAM) were upregulated in malignant epithelial cells. At the same time, nearly all CLDN family genes were involved in the cell adhesion molecules pathway and expression in LUAD-derived cells (**Figures S5A, B**). Notably, malignant cells especially expressed both CLDN1 and CLDN4, while CLDN18 was mainly expressed in the non-malignant epithelial cells (**Figures S5A, B**). Additionally, samples from the TCGA database showed CLND1 expression relatively specific to cancer types (**Figure S5C**). The results indicated that LUAD was characterized by a unique role in the cell adhesion molecules pathway. To confirm their expression of CLDN4 and CLDN1, immunofluorescence was performed using the laser scanning confocal microscope (**Figure 4D**). As shown in **Figures S5B**, CLDN1 and CLDN4 as conventional tumor markers were expressed in malignant epithelial cells, while they were nearly absent in non-malignant epithelial cells. Thus, we recognized that CLDN1 or CLDN4 could be the potential therapeutic targets for LUAD. Malignant LUAD cells were found to have abnormal transcriptional regulatory networks using SCENIC analysis. Notably, some transcriptional factors closely related to LUAD tumorigenesis, such as HDAC2, were upregulated in malignant cells. In comparison, the transcriptional activation factors, such as FOXA2 (inhibiting tumor growth), were downregulated in malignant epithelial cells (**Figure 4E**). These data revealed the new regulatory networks controlled by transcriptional activation factors and provided novel insights into the mechanism of LUAD.

Finally, we investigated the interaction between cell subgroups in the TME and the cancer cells using CellChat. The LUAD cells demonstrated higher levels of midkine (MDK) interacting with receptors expressed on the other TME cells (**Figure 4F**). The MDK encoded protein promoted cancer cell growth, metastasis, and angiogenesis. And the MDK interaction with the LRP1 receptor was associated with immunosuppressive macrophage (M2) differentiation (27). These ligand-receptor pairs (including MIF − (CD74 or CXCR4), MIF − (CD74 or CD44), MDK–NCL, and MDK-LRP1) were more frequently occurring in tumors (28). And they served to regulate tumor growth and immunomodulatory processes. These data were similar to previous studies and indicated that abnormal energy metabolism was an important pathway for LUAD progression (29).

### Enrichment and heterogeneous expression profile of fibroblasts in LUAD

**Figure 5:**
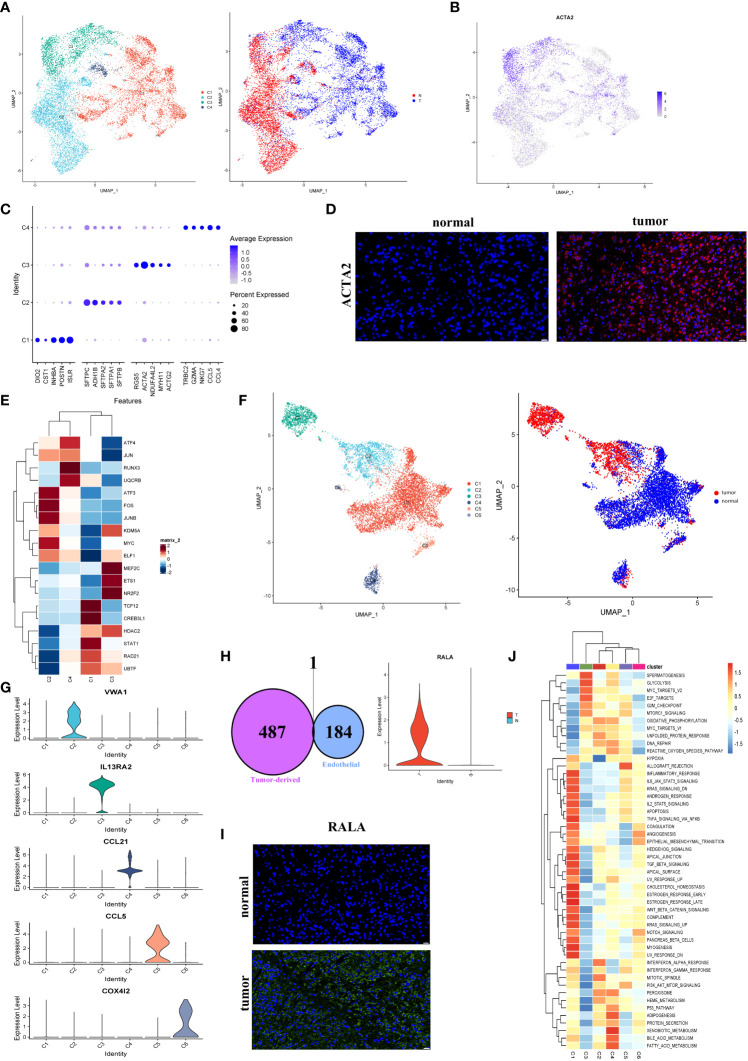
Fibroblasts and endothelial cells demonstrated high heterogeneity in LUAD. **(A)** UMAP plot of four fibroblast clusters and tissue type origin (normal or tumor). **(B)** UMAP plot of the expression level of ACTA2. **(C)** The top five markers of four clusters were shown on the dot plot; color represented expression level, and sizes represented abundance. **(D)** Immunofluorescence of ACTA2 in tissue sections. The scale bar represented 20 µm. **(E)** The heatmap showed the transcription factors’ activity through SCENIC estimated AUC scores in fibroblast. **(F)** The endothelial cells were color-coded (left) for six endothelial clusters and (right) for tissues of normal or tumor origin. **(G)** The marker gene for different endothelial clusters. **(H)** The Venn diagram intersected the endothelial-specific markers and the DEGs between different endothelial cell types (tumor and normal). One overlapped gene was identified (left). The expression level of RALA was visualized by the violin plots (right). **(I)** The immunofluorescence of RALA in LUAD or normal tissue sections. The RALA was upregulated in LUAD tissue. The scale bar represented 20 µm. **(J)** The GSVA estimated the 50 hallmark pathway activities in the different cell clusters.

As demonstrated in **Figure 5A**, fibroblasts were clustered into four subclusters, and most C1 and half of the C3 fibroblasts originated from tumor tissues. As shown in **Figure 5B**, the majority of fibroblasts expressed α-SMA (ACTA2), a conventional marker of fibroblasts. Bubble charts were used to visualize the top five markers of the different clusters (**Figure 5C**). We saw that ACTA2 was highly expressed mainly in C1 and C3. To confirm the phenotype, we stained ACTA2 with immunofluorescence (**Figure 5D**). In addition, RGS5 was known to promote cancer differentiation and metastasis in NSCLC (30), which was also enhanced in C3.

The SCENIC analysis revealed that the transcriptional activity of TCF12, CREB3L1, and STAT1, which were associated with malignant progression, proliferation, and migration, were upregulated in cells from C1 (**Figure 5E**). According to our data, tumor-associated fibroblasts exhibited the promoting tumor growth phenotype.

### Endothelial cells derived from tumors contributed to the progression of LUAD

According to the present study, 8430 endothelial cells were detected from the tumor or normal tissues. Six clusters were identified among these cells (**Figure 5F**). Our subsequent analysis identified each cluster’s markers and showed that most endothelial cells in LUAD (C2) were blood endothelial cells (FLT1+, **Figures 5F**, **S6A**). Four clusters, including C1, C5 (CCL5^+^), C4 (CCL21^+^), and C6 (COX4I2^+^), were enriched in normal tissues, respectively. While C2 (VWA1^+^) and C3 (IL13RA2^+^) were nearly derived from tumor tissues (**Figures 5F, G**). Numerous reports had shown that the IL13RA2^+^ endothelial subgroup played important roles in immunosuppression in the LUAD TME (31). No marker was detected in C1 cells, which were mainly derived from normal tissues. Nevertheless, their role in the biological process couldn’t ignore.

To further identify biomarkers associated with tumors, the endothelial cells’ marker genes and the upregulated genes in tumor-derived endothelial cells were overlapped. Then we obtained one gene, RALA (**Figure 5H**; **Figures S6B, C**). Almost all cancer types showed an increase in RALA, which was well-known as an endothelial activation marker (32) (**Figure 5I**). However, the TCGA bulk RNA-seq data showed the expression of RALA to be downregulated in LUAD (**Figure S6D**). Despite this, our single-cell RNA sequencing analysis further revealed that the RALA was the tumor-derived endothelial cell marker in LUAD. It could serve as a potential therapeutic target for LUAD.

Based on the result of the GSVA pathway analysis on Hallmarker sets, it was found that two endothelial cell clusters (C1 and C3) appeared prominent and significant differences from each other (**Figure 5J**). Remarkably, C1 cells exhibited an enriched inflammatory response. Instead, C3 exhibited an enriched immune inhibitory pathway, which indicated that a high suppression phenotype was derived from the cells from C3. Furthermore, an increased proliferation phenotype (MYC pathway) was strongly enriched in C3. The above observations confirmed that tumor-derived endothelial cells contributed to the progression of LUAD.

### Antitumor immune cells were associated with advanced prognosis in LUAD

**Figure 6:**
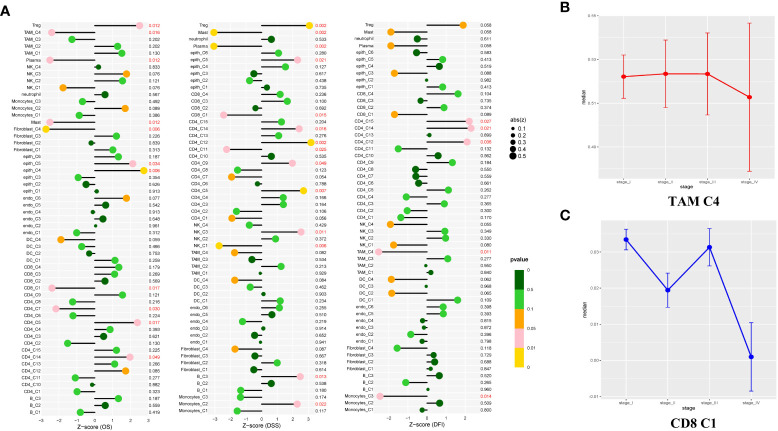
Study of the prognostic effect in the TCGA cohort. **(A)** The lollipop plot showed the correlation between the different cell clusters and disease-free interval (DFI), disease-specific survival (DSS), or overall survival (OS). The risk was measured in Z score with a Cox regression model. The clusters with better outcomes (p < 0.05). **(B, C)** Line charts showed TAM C4 and CD8 C1 enriched in early-stage LUAD tissues, but showed opposite properties in the late-stage. Measured with the one-way ANOVA test.

As shown in **Figure 6A**, the two clusters (macrophage C4 and CD8^+^ T cell C1) were associated with better overall survival (OS), disease-specific survival (DSS), or disease-free interval (DFI) (*p* < 0.05). The proportion of these cells in the LUAD was significantly lower compared with that of the normal tissue (**Figure 1E**). Based on these results, we deduced the M0-like macrophages and CD8^+^ C1 cells with normal functions may be involved in the antitumor function of the TME in LUAD. NK cells C1 and C3 had a better DSS (*p* = 0.006, *p* = 0.011), implying that unidentified mechanisms may contribute to the antitumor process in LUAD *via* NK cells.

Notably, a significant reduction in the proportion of the macrophage C4 and CD8^+^ T cells was significantly decreased in advanced tumor stage samples (**Figures 6B, C**). To determine the independent prognosis of macrophage Cluster 4 or CD8+T Cluster 1, we performed multivariate Cox regression analysis for OS, including clinical features (Stage, T, M, N) and the estimated proportion of cell-types (**Figure S7**). We found only the macrophage Cluster 4 was an independent predictor for better OS. Our research demonstrated that macrophage C4 and CD8+ T cell C1 exerted antitumor activities in LUAD. The number of these two clusters decreased as the LUAD progressed, confirming their antitumor function.

## Discussion

Nowadays, the treatment of LUAD is still a challenge to clinicians. Although immunotherapy is considered a first-line treatment for patients with LUAD, the effectiveness and drug resistance of anti-PD-1 treatment remain notable problems despite the possibility of benefit to a few patients. According to a recent study, both the tumor-infiltrating cells and the cancer cells contribute to therapeutic non-response or drug resistance (33), and the underlying mechanisms need to be closely investigated. In the present study, our analysis of multiple LUAD scRNA-seq datasets unveiled an in-depth analysis of immune and unimmune cells, and we also utilized the immunofluorescence technique to identify the markers of the crucial cell subgroups in clinical cohorts. In the present work, the tumor-specific altered pathways, a series of novel cell subgroups, and novel transcriptional activation factors-driven regulatory networks were identified in LUAD. The results would provide novel targets for prognosis and treatment and contribute to better understanding of intratumoral heterogeneity in LUAD.

Although several observations had been reported for intratumoral heterogeneity, much work still needed to be done due to the highly intricated TME in LUAD. Several findings need to note. First, M0-like macrophages (C4) exhibited KIAA0101^+^FABP4^-^ phenotype, M2a-like macrophages (C1) exhibited KIAA010^-^FABP4^+^ phenotype and M2b-like macrophages (C2) exhibited the ATP5F1E^+^MMP12^-^ phenotype, while another ATP5F1E^+^ MMP12^+^ (C3) subgroup similar to M1 macrophages exhibited pro-inflammatory properties. Notably, in the TCGA cohort, patients in the M0 subgroup had advanced outcomes, while the ATP5F1E^+^ subgroup (C2 and C3) showed the opposite. In the analysis of immune checkpoints and pathways, results indicated that the M2b-like TAMs had immunosuppressive properties in the TME *via* downregulation of Th1 cytokines and upregulation of Th2 cytokines, which could induce a shift from Th1 to Th2 dominance. Through SCENIC analysis, we identified several transcriptional factors (such as JUN) related to the immunosuppressive properties of LUAD, and we firstly found that JUN could be a novel immunotherapy target in LUAD.

Second, we found that the exhausted CD8^+^ T cells were highly enriched in LUAD (C2, C3, and C4), whereas the C1, mainly derived from normal tissue, showed a better prognosis in the TCGA cohort. This result was consistent with the study that the infiltration of exhausted CD8^+^ T cells contributed to a worse prognosis in recent studies (34). Pseudotime and differentiation trajectory analysis revealed the T cell exhausted in LUAD and showed the signaling pathways involved in this process. We deduced that it may be possible to reverse T cell dysfunction by intervention in these pathways to revive CD8^+^ T cells against tumor activity (such as TIGIT, TNFRSF9, CTLA-4, LAG3, PD-1), and this approach maybe represented new strategies for immunotherapy against LUAD. Previous studies had demonstrated that TGF- β was highly expressed in LUAD, which could block the efficacy of PD-1 and promote tumor growth and metastasis, which was associated with poor prognosis (35–37). This was consistent with the high expression of TGF- β in exhausted CD8+ T cells (C2) in our study. Therefore, it was suggested that simultaneous blockade of TGF- β and PD-1 signaling pathways would obtain a better antitumor effect. Furthermore, we discovered novel transcriptional factors alterations FLI1, TBX21, XBP1, and MAFF that may contribute to the exhaustion of T cells. These findings would further enhance our understanding of the LUAD pathological condition Based on our deconvolution results, we found that the patients with a high proportion of macrophage C4 exhibited better clinical outcomes. Meanwhile, CD8^+^ C1 with the activated T cells enriched was related to a better prognosis. In contrast, the M2b polarization and T cell exhaustion may gradually increase from low to high grades of LUAD, which implied that M2b polarization and T cell exhaustion played a critical role in LUAD progression. Because immune checkpoints mediated M2b polarization and T cell exhaustion, it was confirmed that blocking immune checkpoints provided a credible approach to LUAD intervention. Consequently, we further confirmed the important role of exhausted T cells in LUAD in this study.

Third, we demonstrated abnormal energy metabolism in LUAD malignant cells. We found LUAD tumorigenesis was significantly correlated with the adhesion molecule pathway and abnormal energy metabolism, which had been rarely mentioned before. Notably, the abnormal adhesion molecule pathway was found in malignant epithelial cells, which was poorly reported up to date and worthy of further in-depth study. Therefore, our study proposes a family of adhesion molecules, i.g. CLND1 and CLND4 as novel therapeutic targets in LUAD treatment. Then, we demonstrated the majority of fibroblasts expressed α-SMA (ACTA2) driven from tumor tissues. And, we further found the expression of RALA was specifically upregulated in endothelial cells driven from tumor tissues. It is worth noting that RALA was shown downregulated in LUAD based on the TCGA bulk RNA-seq data, while almost all other cancer types showed an increase in RALA. Hence, it was for the first time revealed that targeting the RALA in tumor endothelial cells maybe a potential therapeutic target for LUAD.

## Conclusion

Our study revealed immune and non-immune cell subtypes and type-specific gene expression in TME, and shed light on novel therapeutic strategies *via* multicenter scRNA-seq datasets analysis and verification in our clinical cohorts.

## Data availability statement

The original contributions presented in the study are included in the article/**Supplementary Material**. Further inquiries can be directed to the corresponding authors.

## Author contributions

Writing—original draft preparation, TF. Writing—review and editing, ZG and JL. Data curation, LZ. Software, ZG. Resources, TF, ZG, and LZ. Visualization, DN and YZ. Supervision, ZG, BZ, ZZ, and LZ. Funding acquisition, LZ, XH, NP, BL and HF. Providing clinical information, XH. Collection of raw data, NP, BL and HF. All authors had read and agreed to the published version of the manuscript.

## Funding

This research was funded by Natural Science Foundation of Shandong Province, China grant number (No. ZR2021MH138), The China Postdoctoral Science Foundation grant number (No. 2016M592142), Wu Jieping Medical Foundation Clinical Research Special Fund (No. 320.6750.2021-2-94), The Scientific Research Fund of Hunan Province Health Commission (No. 202101062143), National Innovation and Entrepreneurship Training Program for College Students (No. S202010545069S) and Innovation and Entrepreneurship Training Program for College Students of Hunan Province (No. 3697).

## Acknowledgments

The author thanks ZG, LZ, and all team members for their cooperation and contributions.

## Conflict of interest

The authors declare that the research was conducted in the absence of any commercial or financial relationships that could be construed as a potential conflict of interest.

## Publisher’s note

All claims expressed in this article are solely those of the authors and do not necessarily represent those of their affiliated organizations, or those of the publisher, the editors and the reviewers. Any product that may be evaluated in this article, or claim that may be made by its manufacturer, is not guaranteed or endorsed by the publisher.
