# Supplementary material for: Immune and non-immune cell subtypes identify novel targets for prognostic and therapeutic strategy: A study based on intratumoral heterogenicity analysis of multicenter scRNA-seq datasets in lung adenocarcinoma
Source: Front Immunol. 2022 Nov 22;13:1046121. doi: 10.3389/fimmu.2022.1046121 (PMC9723329; doi:10.3389/fimmu.2022.1046121)
Supplement: Supplementary file 1 [file DataSheet_1.pdf]

Supplementary Figure 1

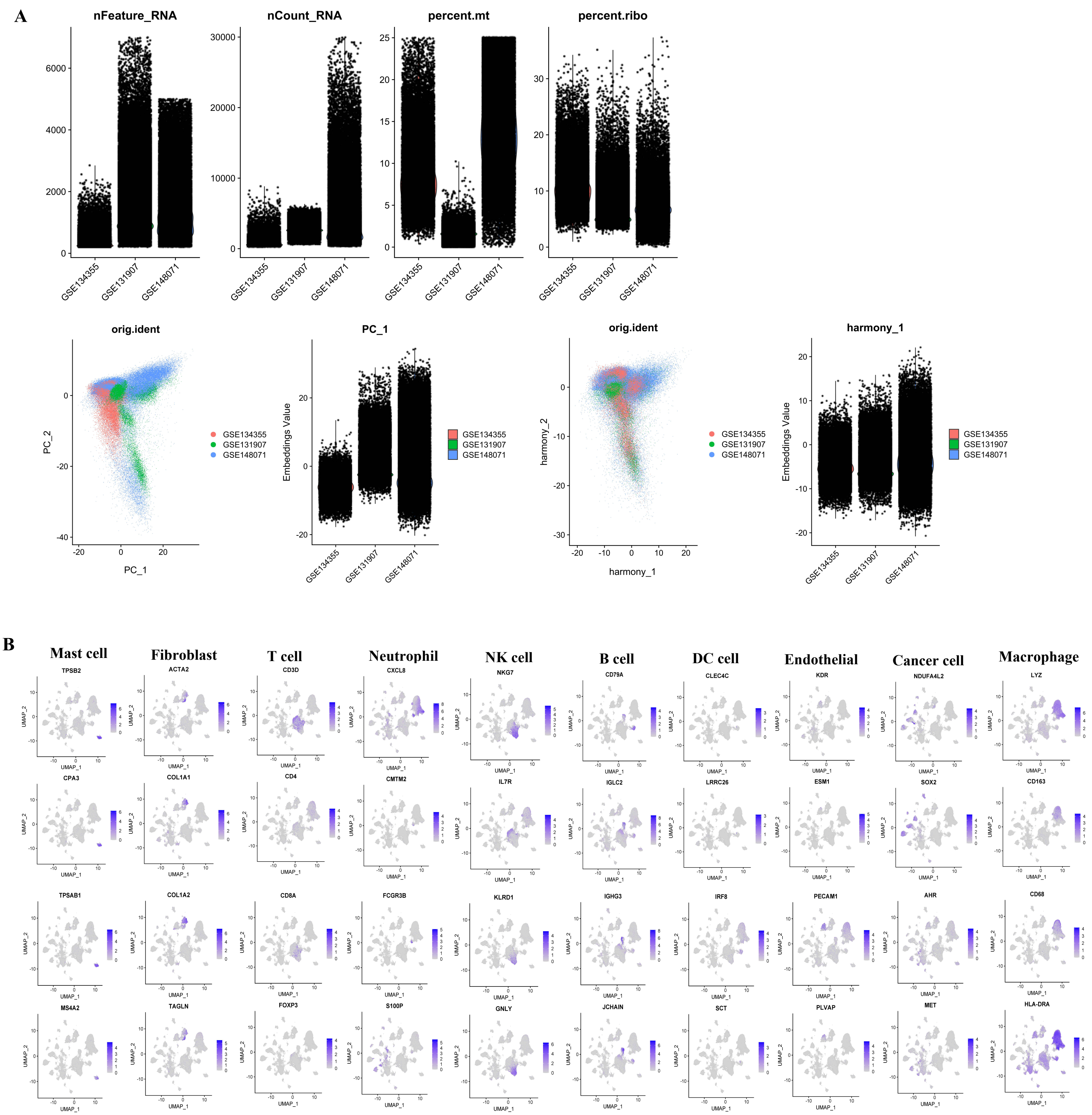

Supplementary Figure 1. (A). The quality control (QC) process and the correct batch effects. **(B)**The Marker genes of major cell types. The four markers of each cell type were visualized in the tSNE plot, respectively.

Supplementary Figure 2

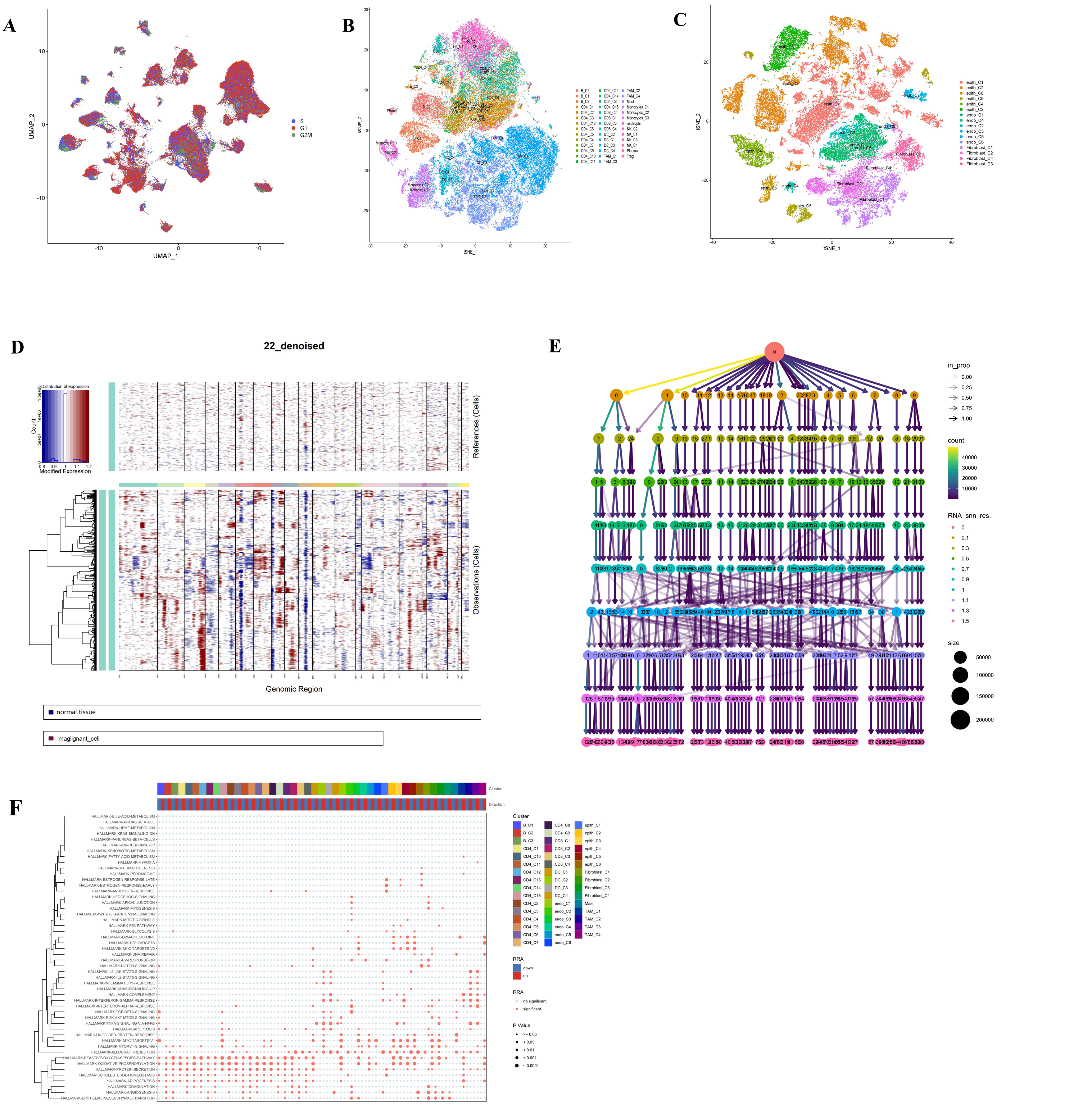

Supplementary Figure 2. (A). UMAP plot colored by cell cycle status. The encircled cell clusters were filtered out. **(B).** tSNE plot of subgroups of CD45+ cells, cell clusters with less than 50 cells were not included in downstream analysis. **(C).** tSNE plot of CD45 subgroups. **(D).** Inferred CNV based on neoplastic cells scRNA-seq divided by patient ID and subtypes. Red means malignant cells, and blue indicates normal tissue. **(E).** Different resolution thresholds from 0-1.5 were visualized. **(F).** The irGSEA result was visualized by irGSEA.bubble function.

Supplement Figure 3

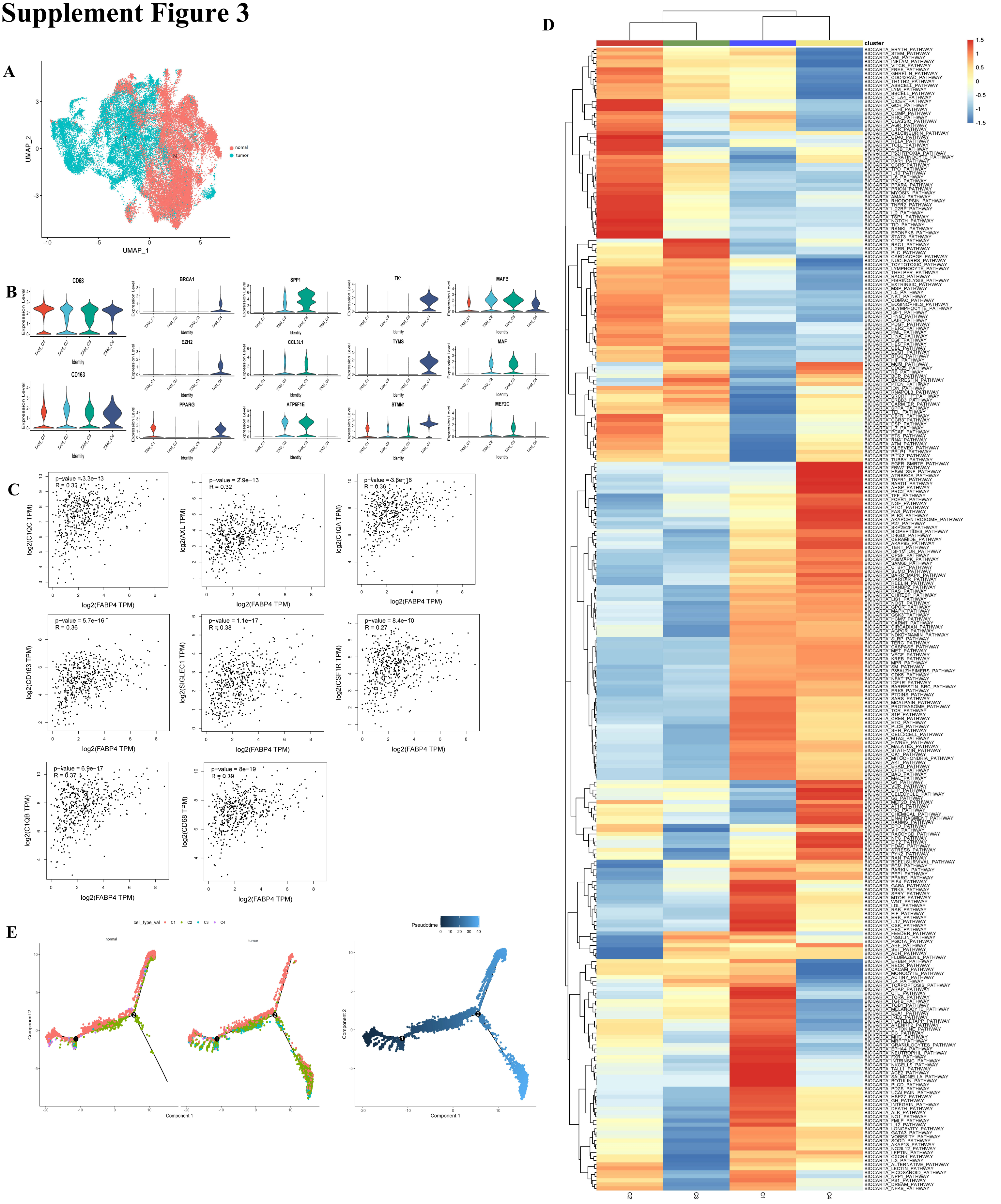

Supplementary Figure 3. (A). UMAP plot of all the normal or tumor tissue of macrophages. **(B).** The top marker genes of C1-C4. **(C).** Correlation between FABP4 and M2-like TAM markers in TCGA. **(D).** Differences in immune-related pathway activities scored with GSVA. Shown were t values calculated by a linear model.

Supplement Figure 4

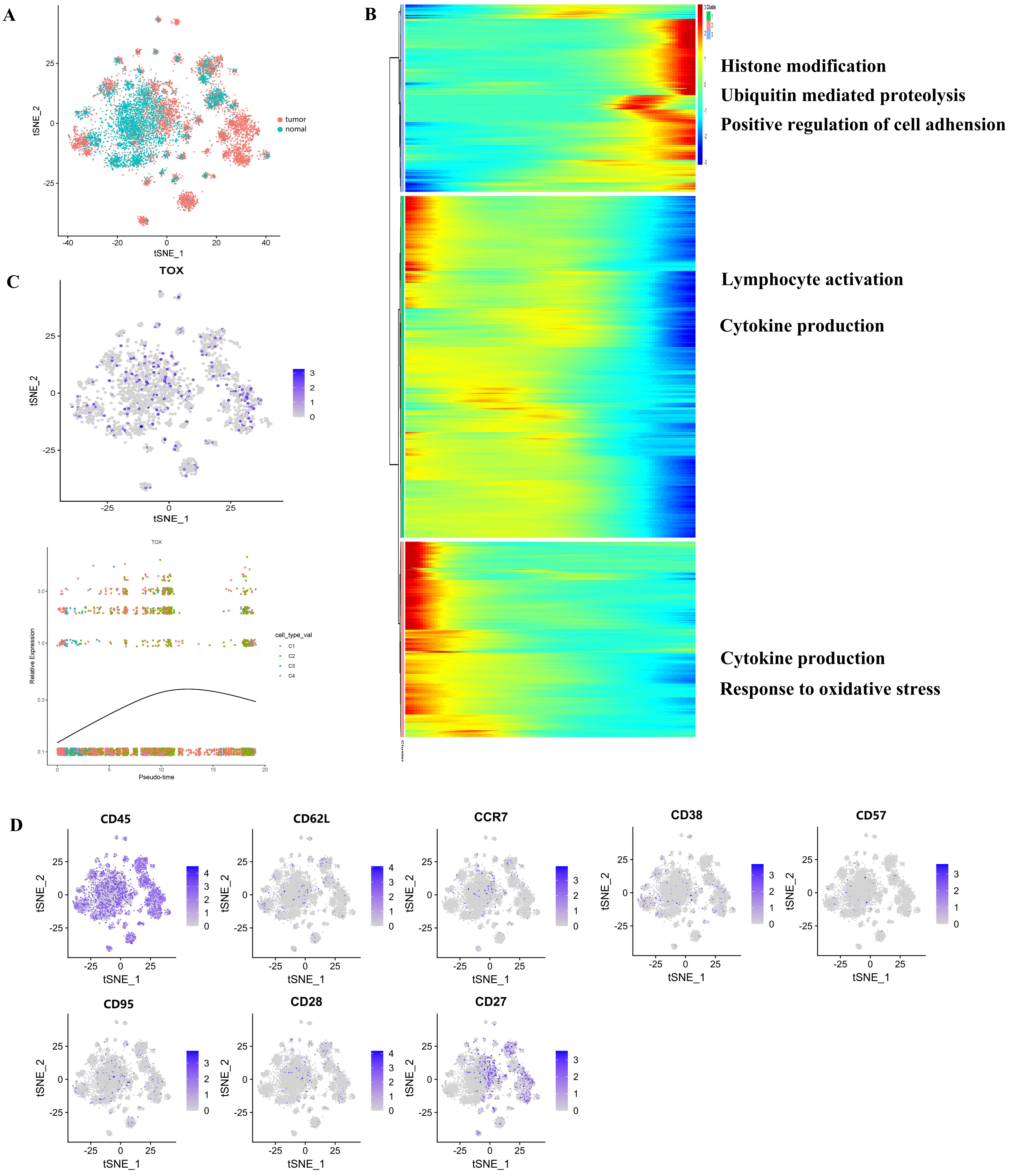

Supplementary Figure 4. (A). tSNE plot of four subgroups of all the normal or tumor tissue of CD8+ T cells. **(B).** Pseudo-time heatmap showed the expression of different genes in the differentiation of CD8+ T cells in LUAD TME. Genes were divided into 3 clusters, and GO enrichment analysis was performed. Enrichment results were shown on the right (FDR < 0.05). **(C).** The transcription factor TOX expression alteration in T cell differentiation. **(D).** The Marker genes of major cell types were visualized in the tSNE plot, respectively.

Supplement Figure 5

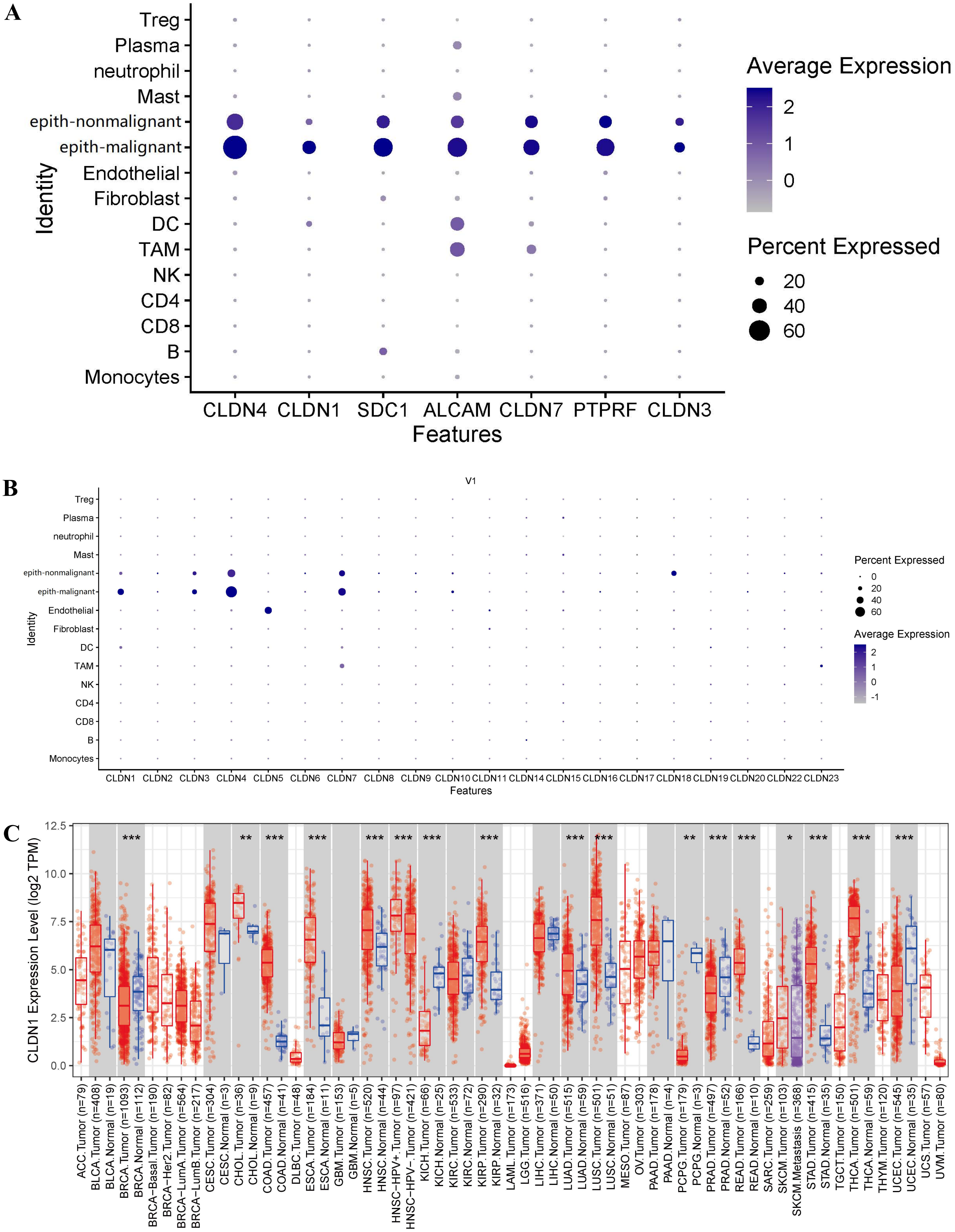

Supplementary Figure 5. (A-B). Expression of selected genes **(A)** and CLDN family members **(B)** in LUAD TME. **(C).** Expression of CLND1 among different cancer types in TCGA.

Supplement Figure 6

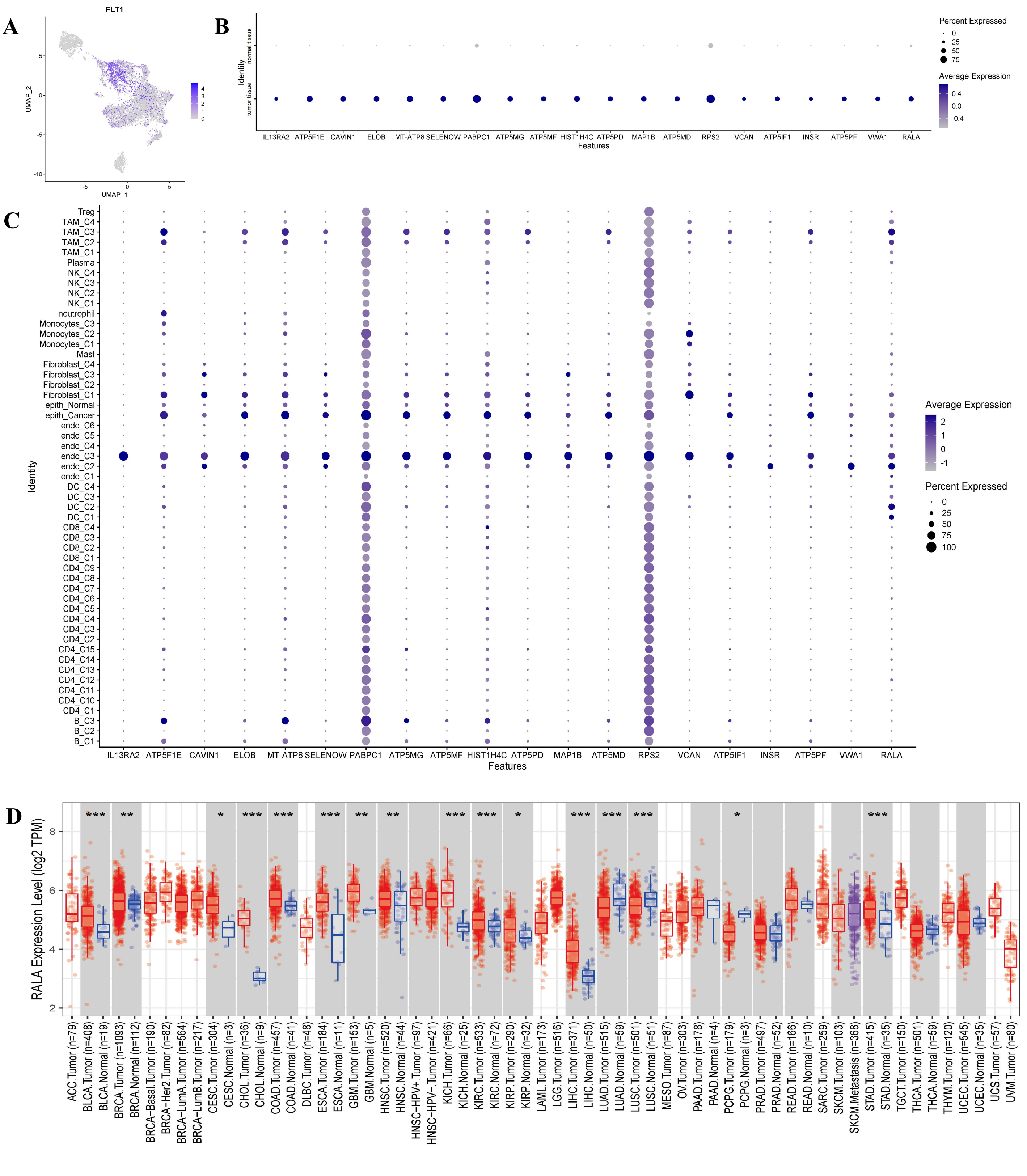

Supplementary Figure 6. (A). tSNE plot of FLTl expression was the marker of blood vessel endothelial cells. **(B).** The upregulated genes in tumor-derived endothelial cells. **(C).** The expression level of endothelial-specific genes. **(D).** Expression of RALA among different cancer types in TCGA.

Supplement Figure 7

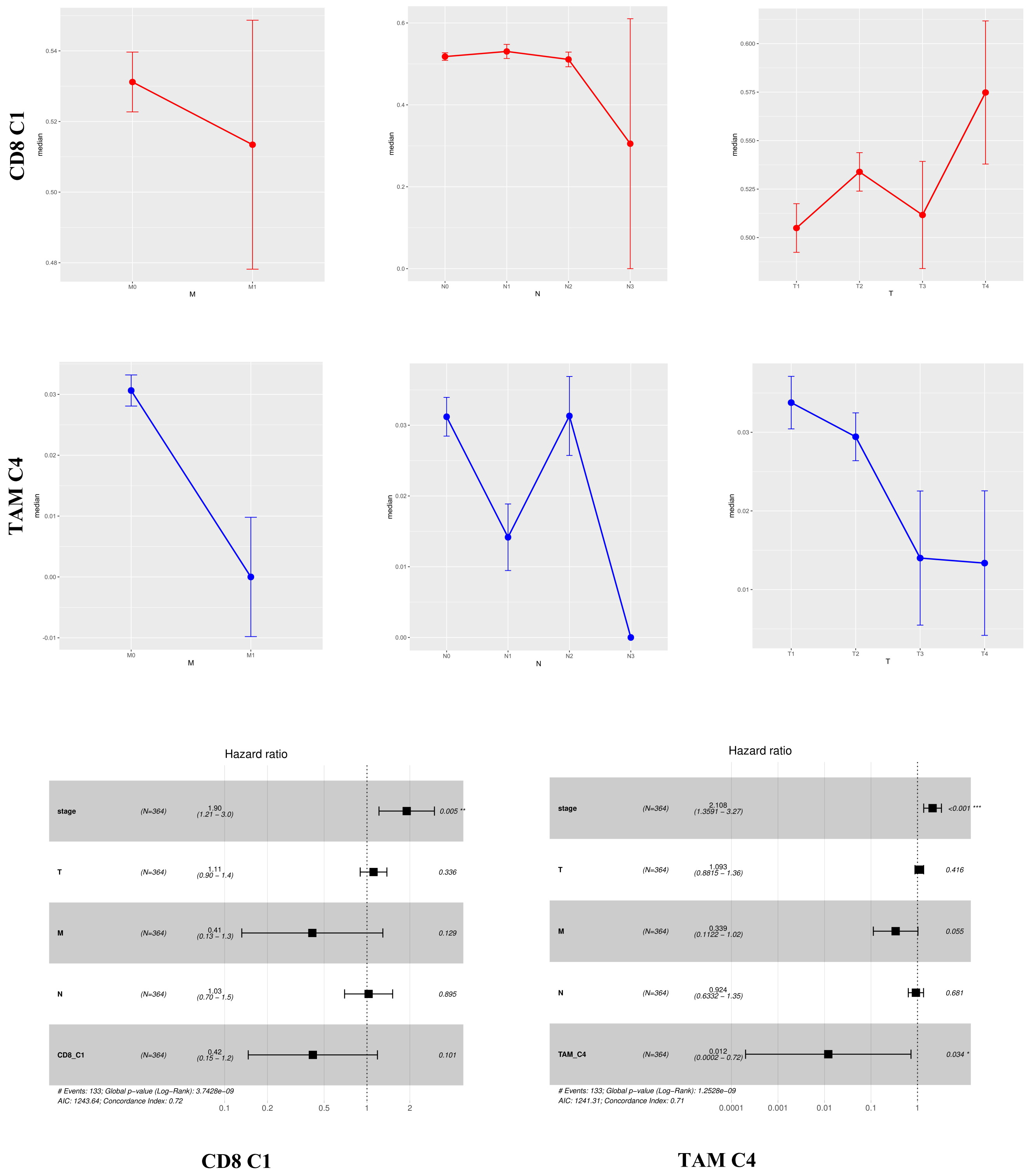

Supplementary Figure 7. Line charts showed TAM C4 and CD8 C1 had opposite properties in the late-stage (up and middle). Multivariate Cox regression analysis in different clinical features (Stage, T, M, N) and the estimated proportion of cell-types (down).
